# A Survey on the Implementation of Analytical Quality by Design in Method Development in the Pharmaceutical Industries of Southeast Brazil: Challenges and Opportunities

**DOI:** 10.1007/s43441-026-00930-2

**Published:** 2026-02-24

**Authors:** Bianca C. Claro, Erica F. Condado, Livia D. Prado

**Affiliations:** 1 Post-graduation Program of Management, Research and Development in Pharmaceutical Industry, Farmanguinhos, Fiocruz, Rio De Janeiro, RJ Brazil; 2 Coordination of Technological Development, Farmanguinhos, Fiocruz, Av.Comandante Guaranys, 447 Jacarepaguá, Rio De Janeiro, RJ 22775-903 Brazil

**Keywords:** Regulatory science, Analytical quality by design, Method development, Control strategy, Survey, Quality management

## Abstract

Analytical Quality by Design (AQbD) is a systematic approach that integrates quality into method design, ensuring robustness, fitness for purpose, and consistency throughout the lifecycle. This study aimed to assess the current scenario, benefits, and challenges of implementing AQbD in analytical method development within the pharmaceutical industry. The methodology included a questionnaire survey applied to pharmaceutical companies, complemented by insights from the literature to contextualize regulatory and scientific challenges. While AQbD offers a promising approach for developing robust methods, its full adoption faces technical, operational, and regulatory barriers. The survey found that although 88.3% of the responding companies are familiar with AQbD, only 50% have begun implementation, mainly in early stages with a focus on tools like design of experiments and risk assessment. Key AQbD components remain underutilized. The main barriers to implementation include a lack of technical resources, qualified personnel, cultural resistance, and unclear regulatory requirements. The literature indicates that AQbD has been widely applied. However, methodological inconsistencies persist. The comparative analysis revealed the alignment between literature and practice regarding benefits but also highlighted gaps in AQbD implementation. By identifying the specific challenges faced by regulated sectors, this study provides valuable insights for regulatory agencies to anticipate future needs, refine guidelines, and promote harmonized standards that ultimately strengthen analytical method quality and ensure consistent product quality throughout the lifecycle.

**Supplementary Information:**

The online version contains supplementary material available at 10.1007/s43441-026-00930-2.

## Introduction

The pharmaceutical industry, constantly evolving and subject to stringent regulations, faces ongoing challenges in ensuring drug efficacy, safety, and quality. The traditional approach to drug development relies on Quality by Testing (QbT), where the quality of drug attributes is ensured through testing, primarily on the final product, based on specifications defined and approved by regulatory agencies. Additionally, the production process is strictly controlled with specifications to prevent quality deviations. When deviations occur, they often result in product disposal without identifying the root cause, due to a lack of understanding of the process, product, and sources of variability [1, 2].

The risk of nonconformities during the production process, scaling issues, and out-of-specification results in quality control using the conventional QbT approach is high [3]. Consequently, there is a growing impetus for the adoption of Quality by Design (QbD) within the pharmaceutical industry to evolve from an empirical process to one that integrates science and risk management [2]. QbD is a strategic approach aimed at embedding quality into the pharmaceutical product from the very beginning, continuing with the development of that quality based on scientific knowledge and the management of risks associated with the manufacturing process [4–6].

The recognition that analytical methods are critical processes with quality requirements has enabled the application of QbD principles to analytical development, leading to the emergence of Analytical Quality by Design (AQbD). The goal of this concept is to ensure that analytical methods are robust, fit for purpose, and consistent throughout their lifecycle [7–9]. AQbD offers a systematic, scientifically grounded strategy, integrating method understanding from the development phase. It encourages multivariate analysis, providing better visualization of the experimental domain, promoting regulatory flexibility, preventing failures, and reducing costs, strengthening analytical quality within the pharmaceutical industry [10–12].

The development of an analytical method using AQbD follows well-defined steps, starting with creating a prospective summary of the performance characteristics, called the Analytical Target Profile (ATP). After this, an analytical technique is chosen to meet these requirements. The next step involves selecting attributes (property related to the analytical technique that must remain within an acceptable limit, interval, or distribution to ensure that the measurement delivers the expected quality) and parameters (operational condition of an analytical procedure that can be adjusted over a range or defined at specific, controlled levels), followed by the development and optimization phase, including univariate or multivariate experiments. The robustness of the procedure is assessed, often by constructing the Method Operable Design Region (MODR), and a control strategy is established [13, 14]. As part of AQbD, it is important to consider measurement uncertainty (characterizes the dispersion of the values that could reasonably be attributed to the measurand). The target uncertainty should be considered in the ATP. With the estimation of uncertainty, a replication strategy (structured approach to determining how many replicates of each step in an analytical procedure are needed to manage sources of variability and ensure that the final reportable value meets the performance criteria defined in the ATP) and guard bands (A deliberate narrowing or adjustment of acceptance limits to incorporate measurement uncertainty) are established to reduce risks of nonconformity [15].

AQbD is gaining prominence in academia, industry, and regulatory bodies. Park et al. (2022) reported a significant increase in scientific publications on the topic from 2012 to 2021 [16, 17]. AQbD has gained recognition from global regulatory agencies, such as the UK’s Medicines and Healthcare products Regulatory Agency (MHRA) [18]. Meanwhile, the United States Pharmacopeia (USP) proposed General Chapter < 1220>, emphasizing the analytical method lifecycle with a continuous approach to development, validation, and ongoing procedure performance verification [19]. This AQbD-based approach aligns with the recently published ICH Q14 guideline, which promotes a science- and risk-based approach to analytical method development for drug quality assessment [13].

Recent regulatory discussions are moving toward a more integrated approach to setting analytical specifications, with updates such as the ongoing consolidation of ICH Q6A and Q6B. These initiatives are part of a broader shift in which specification limits are expected to be supported by a clearer link to patient and product performance, rather than relying primarily on traditional manufacturing acceptance ranges. This direction is consistent with the principles underlying the Analytical Target Profile (ATP), which emphasizes aligning analytical method performance with clinical relevance and intended use [20].

Despite its advantages, the successful implementation of AQbD faces significant challenges, primarily because it is not widely adopted across the pharmaceutical industry and regulatory authorities. Although there are published articles and documents, there is a lack of uniformity in its interpretation, discrepancies in knowledge of stages and definitions, and applications that focus on certain steps at the expense of others. Besides regulatory and methodological barriers, there are also cultural and strategic challenges [21].

This study aims to evaluate the current scenario, how the application of AQbD has been described in the literature, how it is being implemented in industries located in southeast region do Brazil, the challenges faced, and the potential benefits of AQbD in the development of analytical methods in the pharmaceutical industry. Additionally, this study aims to serve as an incentive for the implementation of AQbD in the development of analytical methods by pharmaceutical companies that have not yet started or are in the process of implementing it. By optimizing development and promoting ongoing procedure performance verification of analytical methods, it is possible to reduce risks to consumers (patients), minimizing therapeutic failures or potential toxicity, and decrease risks for the manufacturer (pharmaceutical industry) by reducing the rejection of batches that meet the planned quality.

## Methods

An electronic questionnaire survey was conducted with pharmaceutical companies located in the Southeast region of Brazil to assess their perception of AQbD as an approach for developing analytical methods and to determine the overall implementation landscape of AQbD. The questionnaire is available in the Supplementary Information. The choice of the Southeast region for the survey was based on its representation within the pharmaceutical sector in Brazil [22]. To identify the final population, a search was conducted for national companies with a Good Manufacturing Practices Certificate for Medicines on the Anvisa website, conducted on December 20, 2023. The questionnaire was distributed via email using contact information obtained from company websites and professional platforms such as LinkedIn, and participation was voluntary and confidential. Before accessing the questionnaire, participants were presented with a clear description of the study and what participation entailed, allowing them to evaluate their interest in contributing. The Informed Consent Form (ICF), the questionnaire, and the project were submitted to the Research Ethics Committee via the Plataforma Brasil. The project was assigned the CAAE number: 78792424.8.0000.5262. The approved substantiated opinion was received under number 6.793.928.

This self-administered and confidential questionnaire was sent to companies and collaborators via email addresses obtained from various platforms, such as company websites and LinkedIn. The only response option was through the REDCap platform. Three attempts were made to obtain responses via email. Before accessing the questionnaire, participants viewed an introductory page explaining the purpose of the study, data handling procedures, and their rights as participants. Only those who agreed electronically to these terms could proceed. They were also given the option to download a copy of the ICF and their responses upon submission.

The questionnaire contained 19 questions divided into three sections: the first section is the ICF, the second involves general information about the company, and the third refers to conceptual aspects of AQbD and its implementation. The basis for selection of participating companies were: being a national company located in the Southeast region of Brazil (Espírito Santo, Minas Gerais, Rio de Janeiro, and São Paulo) with a valid Good Manufacturing Practices Certificate. Companies with a certificate line for biological medicines, non-sterile cryogenic liquids, and medicinal gases were excluded from the study. Additionally, those who did not accept the ICF and/or withdrew the consent during or after the questionnaire were also excluded.

For the analysis of the data obtained from the questionnaires sent to pharmaceutical companies, a descriptive quantitative approach was adopted, aligned with consolidated methodologies for survey research [23], using simple statistics for the processing and interpretation of responses. The data were organized and analyzed using Microsoft Excel software, where screening was performed to exclude duplicate and incomplete responses, ensuring the consistency of the analyzed dataset. Categorical variables, such as company type, presence of Research and Development (R&D) department, type of medicine developed, knowledge and degree of AQbD implementation, were evaluated using absolute and relative frequencies, with graphical representation and tabulation to facilitate the visualization of the results. To investigate the relationships between categorical variables (company type × presence of R&D, company type × adoption of AQbD, presence of R&D × adoption of AQbD, and use of AQbD-related tools × implementation stage), non-parametric inferential analyses were performed using the Chi-square (χ^2^) test, with a significance level of *p* < 0.05. Open-ended responses were evaluated through descriptive analysis. This approach allowed the identification of patterns and gaps related to AQbD application in the responding companies, integrating the results with secondary data from the scientific literature and institutional reports from the national pharmaceutical sector.

In order to integrate the data from the scientific literature with the results obtained through the questionnaire, a narrative literature review was conducted on the application of AQbD in the development of analytical methods. The guiding question was: “How has AQbD been applied in the development of analytical methods?” To answer this, searches were performed in the Web of Science and PubMed databases, combining keywords such as AQbD or Analytical Quality by Design, and Pharmaceutical. The searches were conducted in March and April 2024 and adhered to specific inclusion criteria. The inclusion criteria were: original articles published between January 2023 and April 2024, in either Portuguese or English. The exclusion criteria was review articles. This timeframe was selected to ensure the data reflected the current state of AQbD implementation. Initially, titles and abstracts were reviewed to assess relevance to the topic, and duplicate articles were excluded. The literature review conducted in this study was narrative in nature and therefore subject to inherent constraints, including the restricted search timeframe, the choice of databases and languages, and the exclusion of review articles. Its purpose was to provide contemporary context for the survey findings rather than to generate comprehensive or quantitative conclusions,.

## Results and Discussion

### Industry Perspectives

#### General Information About the Companies

The questionnaires were distributed between November 2024 and January 2025 to a total of 142 pharmaceutical companies. Of the 77 initial responses received, 16 were excluded due to duplication (multiple respondents from the same company) or incompleteness, resulting in 60 valid responses (a response rate of 42.3%).

**Fig. 1:**
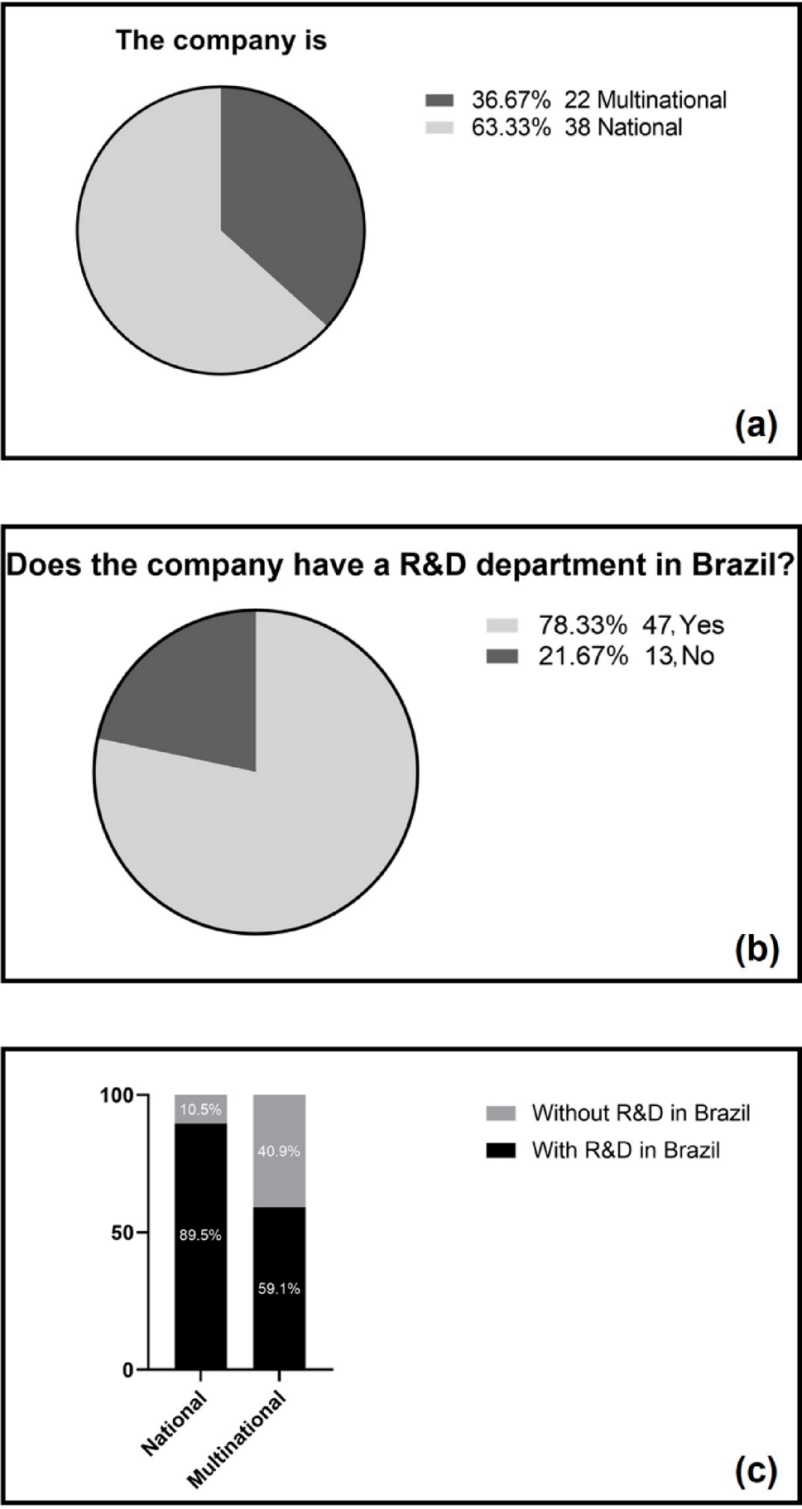
**a** National or multinational participating companies, **b** presence of R&D sector in companies and **c** relationship between company type and the presence of R&D in Southeast Brazil. Percentages are based on valid responses (*n* = 60)

The first evaluation was the distribution between multinational and national companies. Of the 60 responding companies, 22 are multinational, while the majority are nationally operating companies (Fig. 1a). This reflects the predominance of national companies in the Brazilian pharmaceutical sector, where 72% of the 411 pharmaceutical companies registered with ANVISA are locally owned [24]. The proportion of national companies in the sample (63%) is therefore broadly aligned with this national landscape, supporting the contextual representativeness of the dataset. Another relevant aspect analyzed was the presence of a dedicated (R&D) department within the participating companies in Brazil. The results, presented in Fig. 1b, show that majority of companies reported having a specific department for R&D activities. This result highlights the growing recognition of innovation as a competitive differentiator in the national pharmaceutical sector [25].

When analyzing the relationship between company type and the presence of R&D in Brazil (Fig. 1c), it is observed that 89.5% of national companies have an R&D department in Brazil. In the case of multinational companies, the proportion is lower: 59.1% have R&D operations in the country, while 40.9% do not have this department. The results showed a statistically significant difference (χ^2^ = 7.5783; *p* = 0.0059), confirming the association that, in Brazil, national companies are more likely to invest locally in research and development, while multinational companies may concentrate these activities in their home countries. This inference is supported by data from the literature indicating that multinational companies tend to centralize their R&D activities in their home countries or global innovation hubs, relegating their subsidiaries in emerging countries to manufacturing and quality control. In contrast, national companies, which face greater competitive local pressure and limited access to external technologies, tend to internalize R&D as a differentiation and survival strategy in the market [25, 26].

**Fig. 2:**
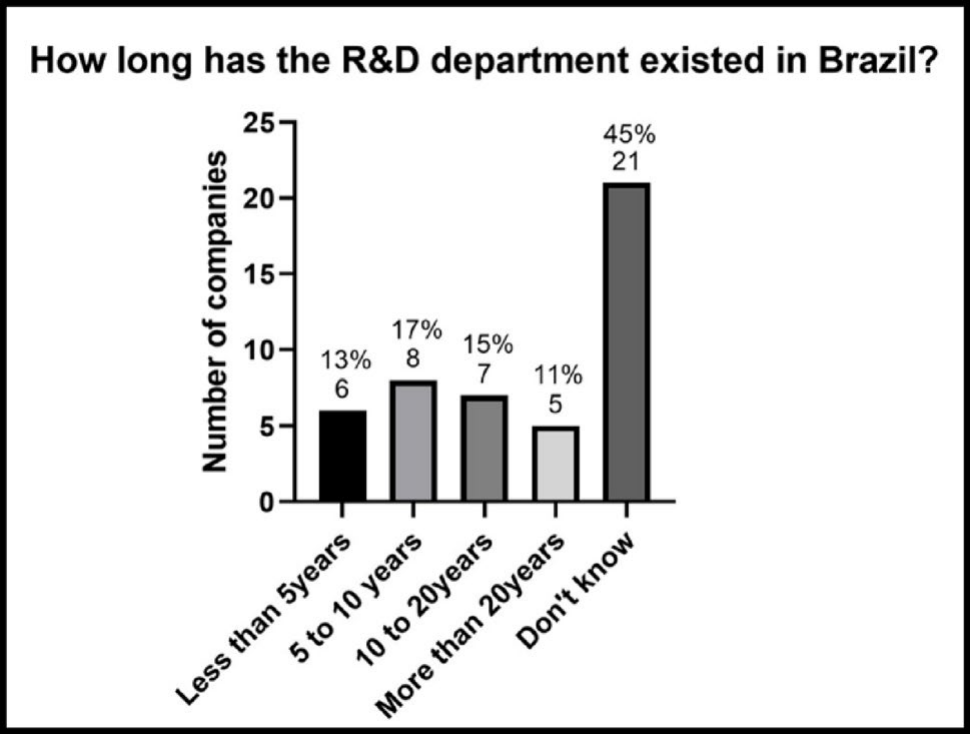
Duration of the R&D sector in Brazil. Percentages are based on companies that reported having an R&D sector (*n* = 47)

As for the duration of the existence of R&D departments in Brazil, there is considerable variation. To facilitate the visualization and analysis of the data, responses to the open-ended question ‘How long has the R&D department existed in Brazil?’ were grouped into ranges, as shown in Fig. 2: less than 5 years, 5 to 10 years, 10 to 20 years, more than 20 years, and ‘I don’t know’. Among the 47 companies with R&D departments, 13% stated that the department has been in existence for less than 5 years, 17% indicated a period of 5 to 10 years, 15% reported having an R&D department for 10 to 20 years, and 11% reported over 20 years of activity. However, a significant number of companies (45%) stated that they did not know how long the department had been established, and 13 companies did not answer this question. This may indicate a lack of familiarity with this information and suggests that, since it is a question that relies on respondents’ recall, the data obtained could be inaccurate.

**Fig. 3:**
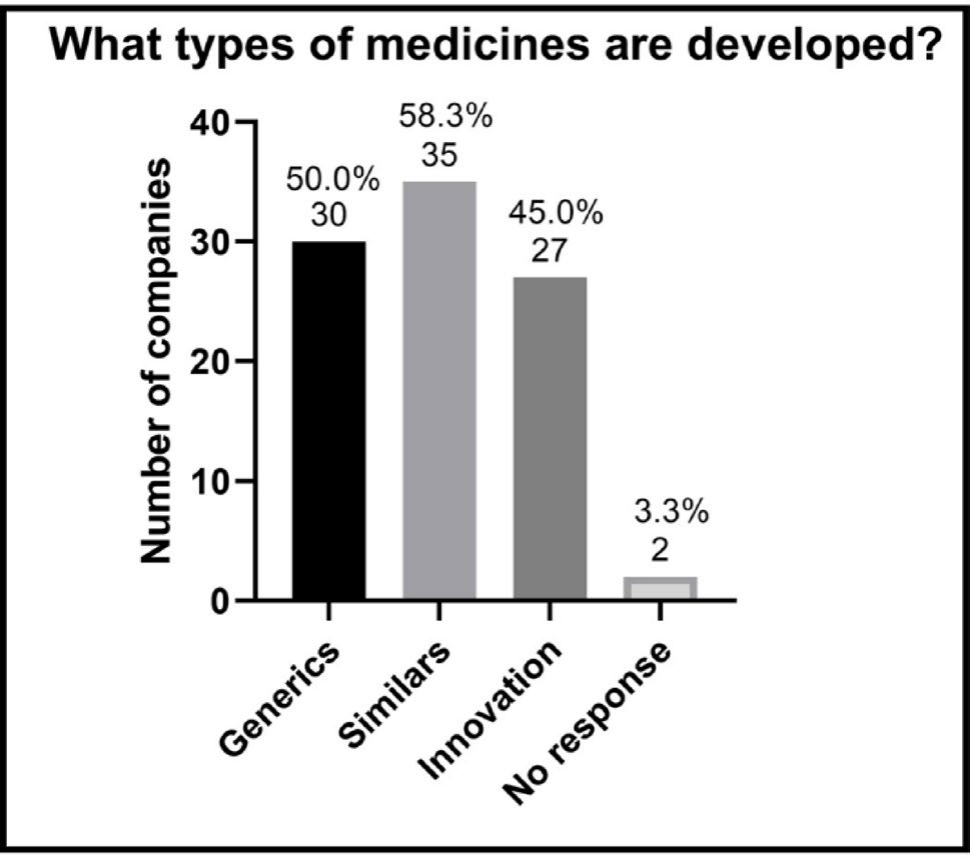
Type of medication developed by companies with R&D in Brazil, according to regulatory strategy. Percentages are based on valid responses (*n* = 60)

In addition to organizational characteristics, the type of medicines developed by the participating companies was also analyzed (Fig. 3). It was observed that the majority of companies work with similar (non-biologic) drug products, with a significant proportion also developing generic and innovative medicines. It is worth noting that respondents from each company were able to indicate more than one category, which reveals the diversification of their development lines.

In the Brazilian context, this diversity reflects the structural segmentation of the national pharmaceutical market. According to data from Anvisa, the generics and similar (non-biologic) drugs segment is predominantly dominated by national companies, which mainly operate with strategies based on volume and price competition [27]. On the other hand, the development of innovative drugs, especially those with radical innovation, remains under the predominant leadership of multinational companies. The distribution of the types of drugs developed by the participating companies confirms this duality within the sector: most of the production is still centered on generics and similar (non-biologic) drugs, but a significant percentage (45%) also reported engaging in the development of innovative drugs. This finding is significant and may be associated with the strengthening of public policies that encourage innovation, such as economic subsidy programs and technological order mechanisms [26]. However, studies indicate that much of this innovation is still focused on incremental strategies, particularly in improving pharmaceutical forms, release systems, and combinations of already known active ingredients [25, 26]. From this data, it can be inferred that the profile of the participating companies in the study is diverse, encompassing both national and multinational companies, with a strong focus on research and development. This scenario is favorable for the adoption of advanced analytical strategies, such as AQbD, as a large portion of the companies already have structures in place focused on innovation and quality.

### Conceptual Aspects and Implementation of AQbD

**Fig. 4:**
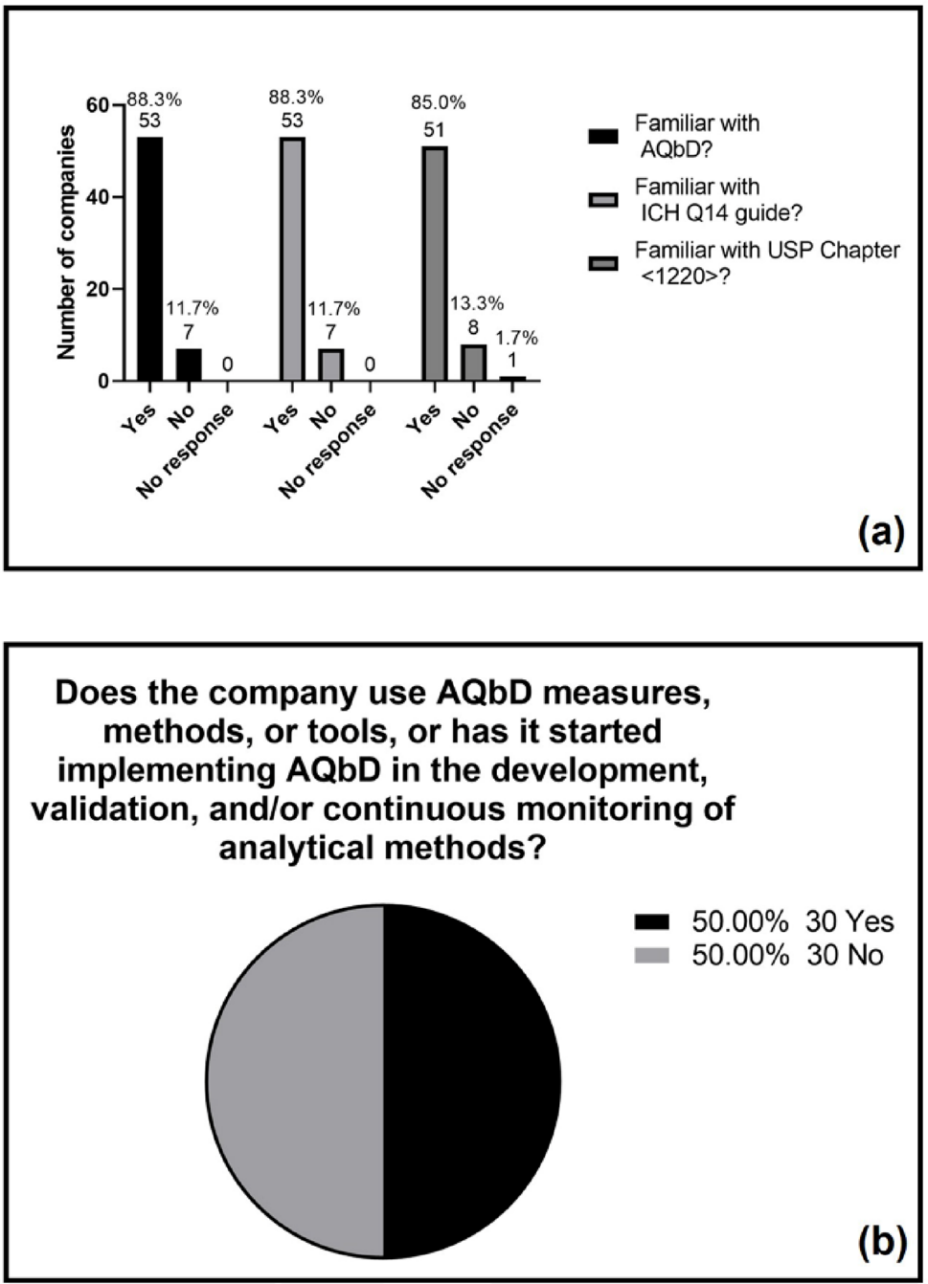
**a** Familiarity of companies with AQbD and its guidelines and **b** adoption of AQbD in analytical methods by participating companies. Percentages are based on valid responses (*n* = 60)

The second part of the questionnaire aimed to investigate the level of knowledge, application, and perception of companies regarding the AQbD approach, as well as to identify the tools and strategies most commonly used in the context of development, validation, and monitoring of analytical methods. Initially, a high degree of familiarity with key AQbD concepts and regulatory references was observed among the companies (Fig. 4a). This familiarity reflects the long-standing use of structured, quality-risk-based analytical development practices in industry, which precede the publication of formal guidelines. Recent regulatory documents have helped consolidate and align these principles, but they are not the source of the theoretical foundation. Thus, the results indicate that AQbD concepts are well disseminated across organizations, supported both by established industrial practice and by more recent regulatory frameworks.

Despite the high level of knowledge about the guidelines and the AQbD approach itself, only half of the companies reported using or being in the process of implementing measures, methods, or tools associated with AQbD (Fig. 4b). This data highlights a gap between theoretical knowledge and practical adoption of the approach.

**Fig. 5:**
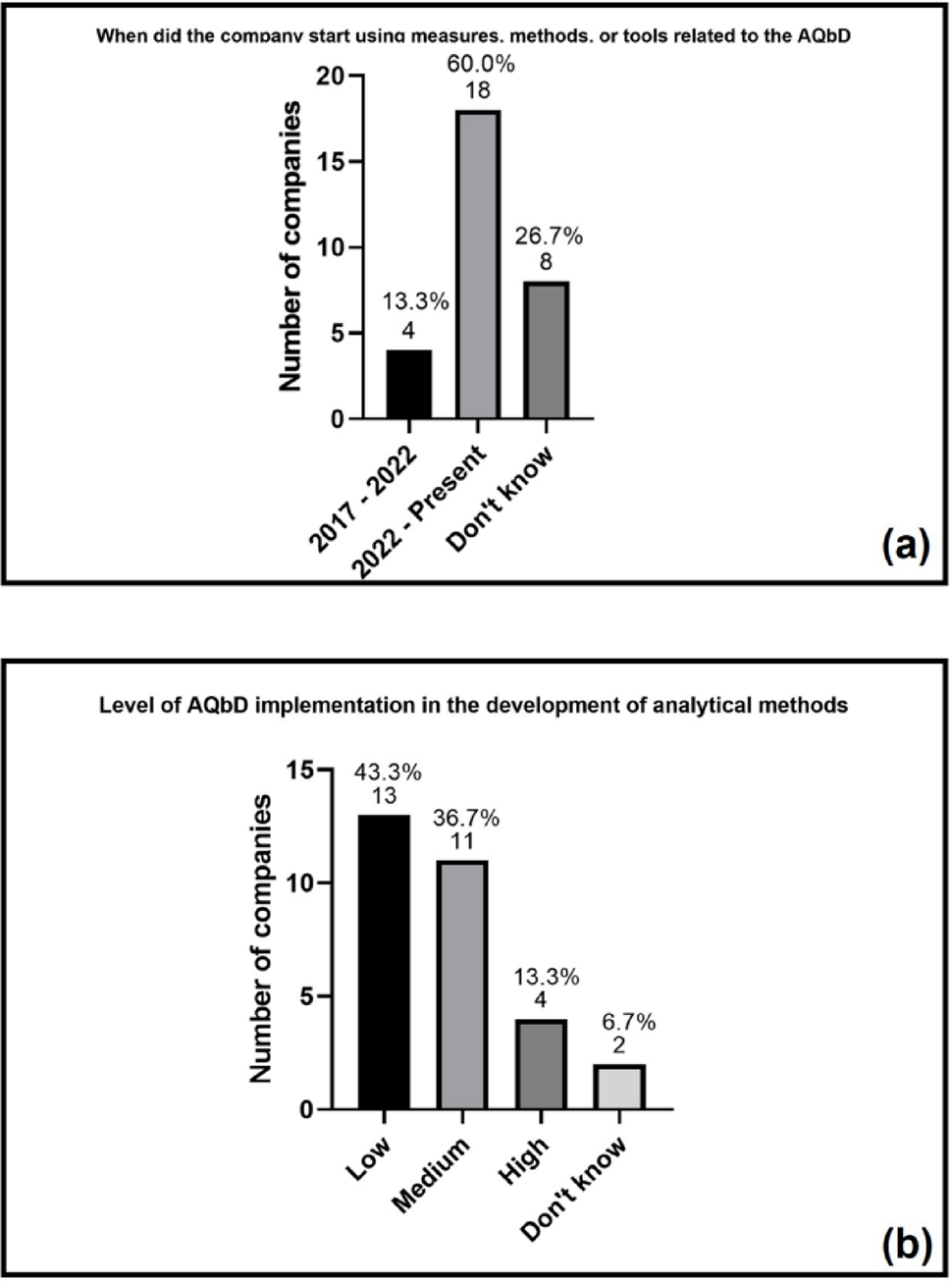
**a** Start period of AQbD implementation and **b** classification of the level of AQbD implementation by participating companies. Percentages are based on companies that reported using measures, methods, or tools from AQbD, or that had started implementing AQbD in the development, validation, and/or ongoing performance verification of analytical methods (*n* = 30)

Among the companies that have adopted AQbD (*n* = 30), the majority began this process after 2022, as shown in Fig. 5a. This suggests a recent trend of adopting the approach, possibly driven by the publication of ICH Q14 in 2023 and the officialization of USP Chapter < 1220 > in 2021 [13, 19]. Only 13.3% of companies (*n* = 4) reported having started implementation between 2017 and 2022, while 26.7% (*n* = 8) stated they could not specify the exact period. This data reinforces the assumption that the move toward AQbD is a recent development in the national landscape, still in the maturation phase.

Regarding the degree of implementation (Fig. 5b) most companies are at an early stage, characterized by a low level of application of the tools prescribed by the approach. Only 13.3% (*n* = 4) considered themselves at an advanced stage, systematically applying this approach in their projects. This result reinforces the idea that, although the topic is being discussed and there is an initial movement toward adoption, most companies are still in the adaptation phase, testing tools and consolidating internal knowledge before reaching systematic application.

No statistically significant association was identified between company type (national vs. multinational) and adoption of AQbD (χ^2^ = 0.53, *p* = 0.466). Similarly, the presence of an internal R&D department did not significantly influence AQbD adoption (χ^2^ = 0.00, *p* = 1.000). These findings suggest that, within the sample, AQbD implementation does not appear to be determined by structural characteristics of the companies, such as ownership profile or the existence of dedicated R&D units. The absence of statistically significant associations indicates that the diffusion of AQbD practices in the Brazilian pharmaceutical sector may be influenced more by organizational culture, maturity of quality systems, or regulatory awareness than by structural attributes such as company type or the presence of R&D. This aligns with international reports showing heterogeneous AQbD adoption patterns that are not strictly determined by company size or ownership model.

The chi-square test indicated no statistically significant association between the use of AQbD-related tools and the reported implementation stage (χ^2^ = 1.85; *p* = 0.397), suggesting that although most companies apply some AQbD tools, their implementation maturity varies independently of tool adoption. This lack of statistical significance indicates that tool adoption alone is not a sufficient predictor of implementation maturity. Many companies appear to apply isolated AQbD elements, such as risk assessment or DoE, without yet achieving a structured or systematic application of the full approach. This pattern reinforces the trend observed qualitatively: companies may have access to tools but remain at early or intermediate stages of AQbD integration due to gaps in training, organizational structure, or regulatory drivers.

Figure 5.

**Fig. 6:**
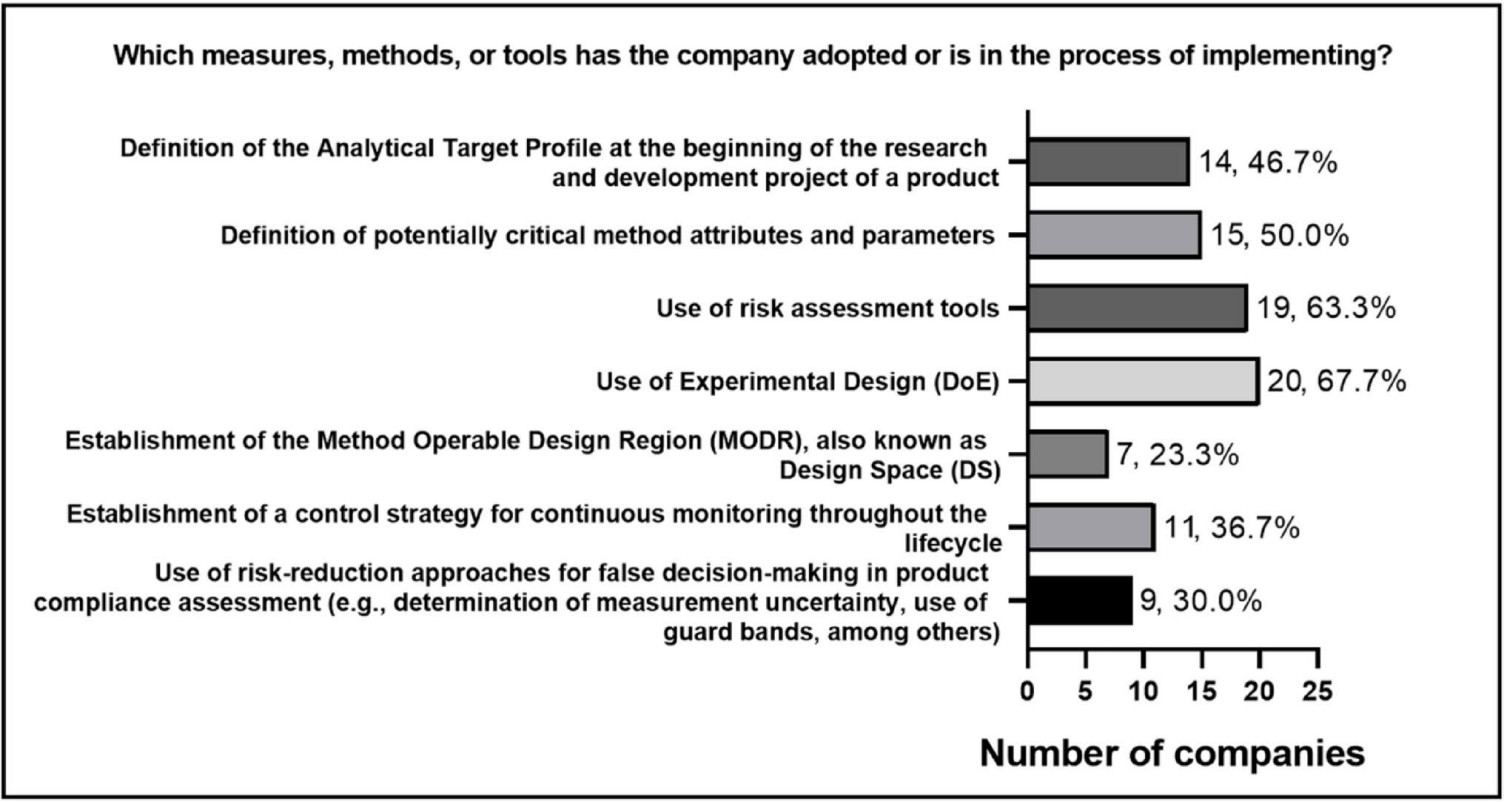
Measures, methods, or tools for AQbD implementation by participating companies

Regarding the tools adopted (Fig. 6) in the context of AQbD, the use of Design of Experiments (DoE) stands out, followed by risk assessment. Defining critical attributes and parameters of the method and defining the ATP also appear frequently, demonstrating a growing concern with method quality, defining the goal and performance to be achieved, and understanding the analytical parameters with the greatest influence on the methods being developed. More advanced and complex practices, such as establishing the MODR and risk reduction approaches for false decisions, were adopted by only 23.3% of companies (*n* = 7) and 30% (*n* = 9), respectively, while continuous monitoring strategies were reported by 36.7% (*n* = 11). These data indicate that companies tend to start implementing AQbD with more familiar tools that have a consolidated technical application, such as DoE, while more complex and integrated stages in the method’s lifecycle still face greater resistance, lack of knowledge, or technical challenges.

**Fig. 7:**
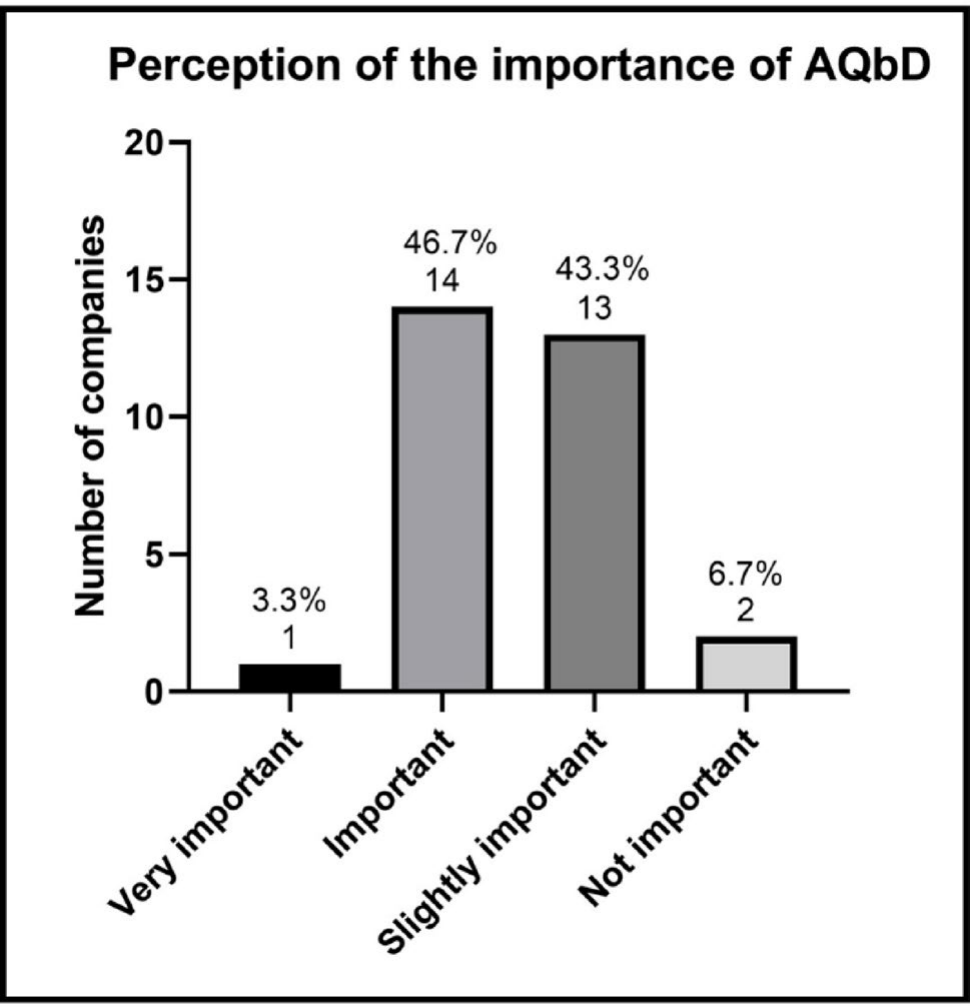
Perception of the importance of AQbD by participating companies. Percentages are based on companies that reported using measures, methods, or tools from AQbD, or that had started implementing AQbD in the development, validation, and/or ongoing performance verification of analytical methods (*n* = 30)

The perception of the importance of AQbD, as measured by the allocation of budget and human resources (Fig. 7), reveals a balanced distribution between companies that consider it “important” and those that classify it as “somewhat important”. Only a minority classified it as “very important,” and one company considered AQbD to be “not important.” This result may reflect the perception that the tangible benefits of the approach are still not sufficiently clear or measurable to justify larger investments in training, implementation of the approach, and purchasing software.

**Fig. 8:**
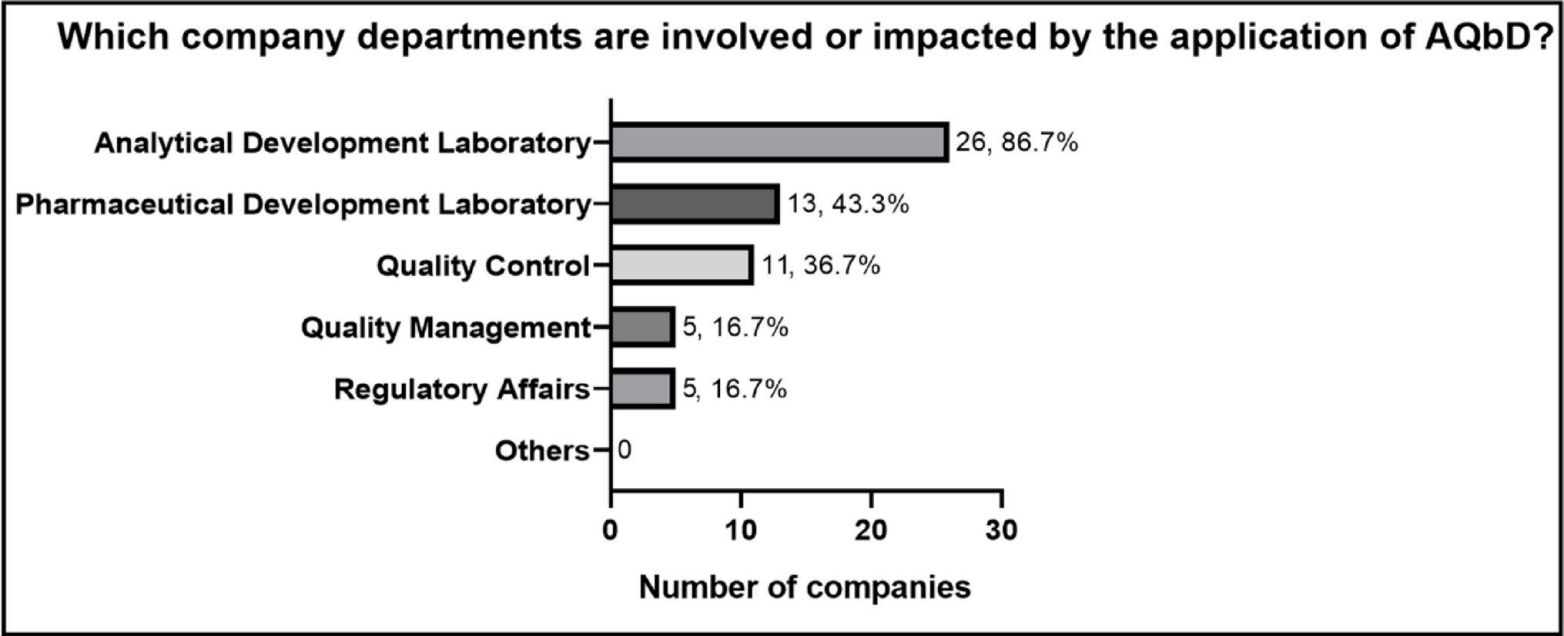
Departments involved or impacted by the application of AQbD by participating companies. Percentages are based on companies that reported using measures, methods, or tools from AQbD, or that had started implementing AQbD in the development, validation, and/or ongoing performance verification of analytical methods (*n* = 30)

The departments most involved in the implementation of AQbD within the organizations are presented in Fig. 8. These include the Analytical Development Laboratory and the Pharmaceutical Development Laboratory, followed by the Quality Control department, and the Quality Management and Regulatory Affairs departments (both with *n* = 5, 16.7%). The predominance of the analytical area as the main driver of AQbD implementation is expected given the nature of the approach.

### Challenges in Implementing AQbD

The final part of the questionnaire focused on identifying the main benefits perceived and the challenges faced by companies in adopting AQbD. This section sought to understand not only the level of practical application of the approach but also the operational and strategic impacts resulting from its implementation in analytical development.

**Fig. 9:**
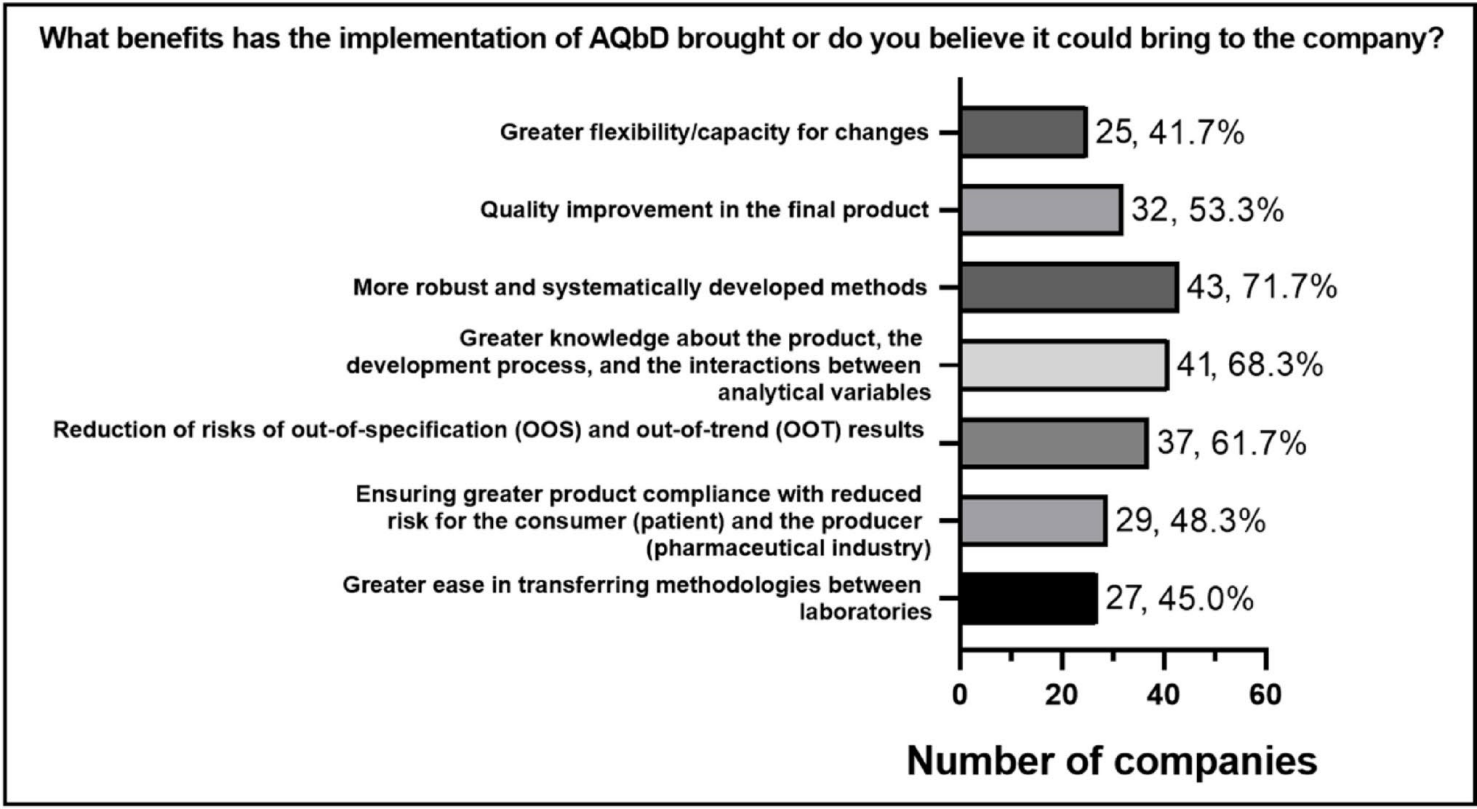
Benefits of implementing AQbD. Percentages are based on companies that reported using measures, methods, or tools from AQbD, or that had started implementing AQbD in the development, validation, and/or ongoing performance verification of analytical methods (*n* = 30)

The data obtained indicate that the main perceived advantage of the current or future application of AQbD is the development of more robust and systematic methods, followed by an increased understanding of the product, the development process, and the interactions between analytical variables, as shown in Fig. 9. These results reflect the core of the AQbD approach, which aims for a deeper understanding of the analytical process to ensure the quality of the data generated, reduce errors, rework, and rejections, making the methods more reliable and reproducible.

Other benefits highlighted included the reduction of risks of out-of-specification (OOS) and out-of-trend (OOT) results, improvement in the quality of the final product, and greater ease in transferring methodologies between laboratories. These aspects are directly related to the philosophy of AQbD, as greater control and knowledge of critical variables and quality attributes allow for the anticipation and prevention of deviations, as well as more secure method transfer. Regulatory flexibility, although mentioned by a smaller proportion, represents one of the most attractive promises of AQbD implementation. While the literature suggests that adopting this approach will allow for method changes within approved limits without the need for new regulatory submissions [1, 11], there remains some uncertainty in the national context regarding how Anvisa will evaluate dossiers [6].

**Fig. 10:**
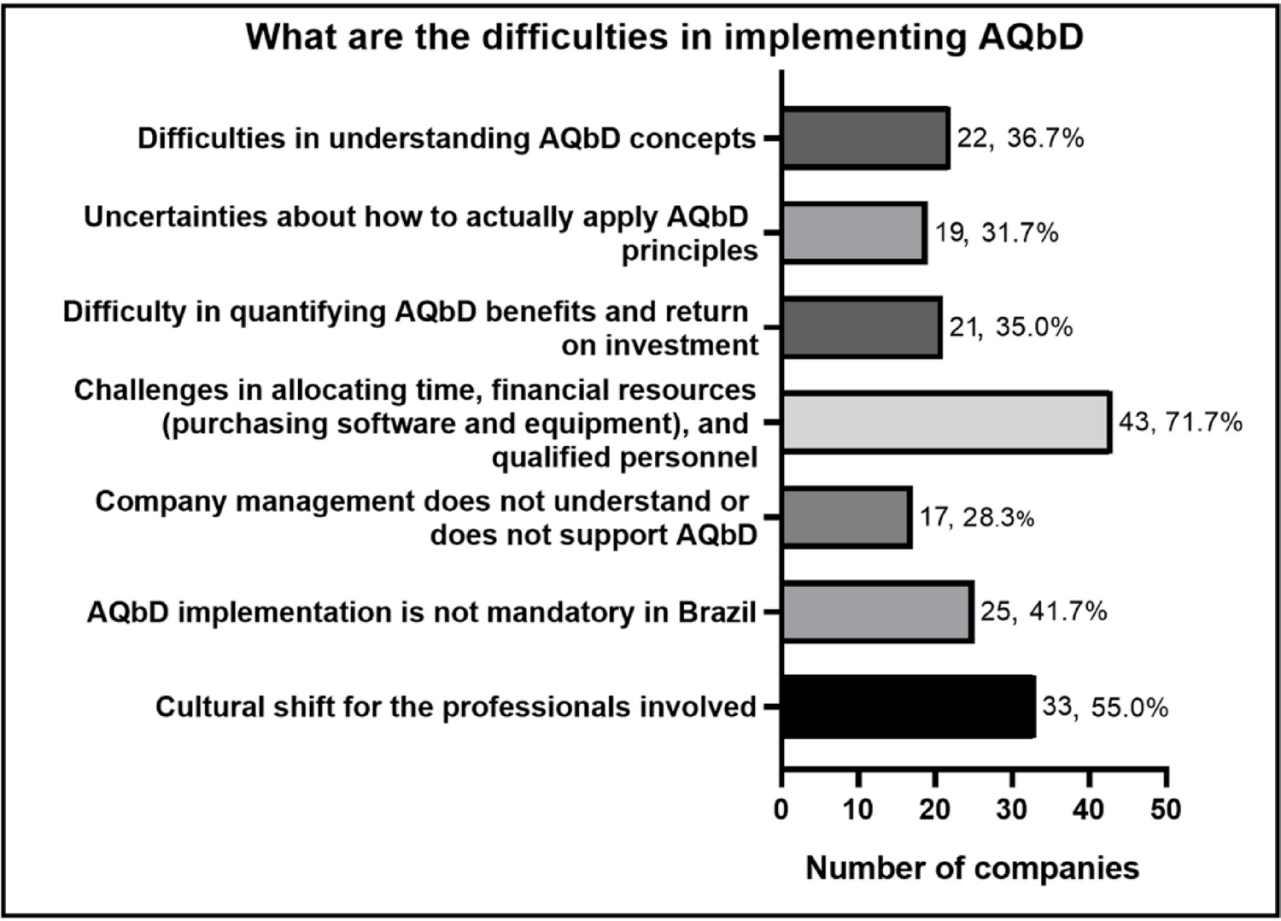
Obstacles faced by companies in implementing AQbD. Percentages are based on companies that reported using measures, methods, or tools from AQbD, or that had started implementing AQbD in the development, validation, and/or ongoing performance verification of analytical methods (*n* = 30)

Despite the recognized advantages, the data also revealed a significant set of obstacles faced by companies in implementing AQbD or that motivated the choice not to implement it, as shown in Fig. 10. The primary barrier identified was the difficulty in allocating time, financial resources, and qualified personnel (*n* = 43, 71.7%). This limitation reflects a common reality in Brazilian pharmaceutical companies, which often face resource management challenges and tight deadlines for development and market launch [28]. Such a scenario makes it difficult to adopt more structured approaches like AQbD, which requires longer-term planning, technical training, and often investments in computational tools and statistical software.

Moreover, 55.0% (*n* = 33) of respondents identified organizational culture change as a significant challenge, highlighting the need for engagement from both professionals and upper management to internalize AQbD as part of analytical development routines. The fact that AQbD is not mandatory in Brazil was also noted as a factor discouraging its adoption, as many companies prioritize efforts to meet minimum regulatory requirements. Furthermore, the difficulty in quantifying the benefits of AQbD and its return on investment hampers convincing management of the medium- and long-term gains that the approach may bring. This likely leads to a lack of understanding or support for adoption of AQbD, as reported by 28.3% of companies (*n* = 17). Additionally, conceptual challenges were also among the most significant obstacles. For 36.7% of participants (*n* = 22), difficulty in fully understanding AQbD concepts was a barrier, while 31.7% (*n* = 19) reported uncertainties about how to properly apply its principles in practice. These findings suggest that, while AQbD is discussed in technical and scientific circles, there are still significant gaps in its operationalization.

The open-ended responses in the questionnaire revealed notable challenges in AQbD adoption by companies. One participant expressed a desire to implement the approach according to ICH Q14 guidelines but pointed out obstacles related to project timelines. They mentioned that AQbD implementation requires revising workflows and additional time for analytical method development, which can result in a competition between method and product development. The participant emphasized that “Analytics is seen as the ‘glasses’ of pharmaceutical development,” indicating the critical importance of analytical methods in the development process. This competition, combined with tight deadlines, may lead to partial application of AQbD, using only certain tools such as DoE, which could result in a misunderstanding that AQbD is merely limited to DoE. The participant also mentioned uncertainty about how Anvisa will evaluate AQbD within companies, noting that, although regulatory flexibility is attractive, full AQbD implementation requires additional preparation and time. Another respondent pointed to the lack of financial resources as a factor contributing to the slow implementation of AQbD in more products within the company’s portfolio.

These observations highlight the practical challenges encountered by Brazilian pharmaceutical companies in adopting AQbD, including financial resource limitations, competition among development timelines, and the necessity for alignment with the regulatory expectations of Anvisa. These factors may influence the decision-making processes regarding the full implementation of the AQbD framework. Such findings emphasize the critical need for the enhancement of technical training for personnel, broader dissemination of AQbD principles, and the establishment of more effective communication channels with regulatory bodies.

### Contributions from the Scientific Literature

#### General Perceptions and Terminologies

The evaluation revealed the use of different nomenclatures and acronyms to describe the elements of AQbD. However, this did not hinder the general understanding of the concepts discussed. This finding contrasts with a review conducted in 2022 [21], which reported a more severe inconsistency between certain acronyms, such as ATP being used instead of AQbD to describe the development of analytical methods, a discrepancy not observed in this literature review. Some authors defined Critical Method Attributes (CMAs) as Critical Analytical Attributes (CAA) [29–32] and Critical Method Parameters (CMPs) as Critical Method Variables (CMV) [29, 31, 32] or Critical Quality Parameters (CQP) [33]. The harmonization of these terminologies represents a specific adjustment that could significantly contribute to clarity and efficiency in reading and interpreting studies.

The evaluation of the study authorship highlighted the application of AQbD not only in academia but also in regulated environments within the pharmaceutical industry. While the majority of papers were authored by academics, university-industry collaboration was observed in two papers developed in Portugal. Chiarentin et al. (2023) involved the University of Coimbra and Basi Indústria Farmacêutica S.A [29]. In Sena et al. (2023), the collaboration was between the same university and Bluepharma—Indústria Farmacêutica S.A [32]. Another study was conducted between a biotechnology-focused pharmaceutical company, Biogen Inc., and the University of Tennessee’s pharmacy department in the United States [34]. The study by Simeoni et al. (2023) was entirely authored by pharmaceutical companies, Novartis Pharma AG in Switzerland and Hexal AG in Germany [35].

Regarding the analytical techniques discussed, as expected, many of the studies analyzed describe the development of analytical methods using High-Performance Liquid Chromatography (HPLC) [15, 30, 31, 33, 36, 37, 38, 39]. The range of factors involved in this technique makes optimization through the traditional approach lead to numerous experiments with an inadequate understanding of key parameters. The AQbD strategy offers a complete interaction of various variables simultaneously in the method’s response, which is highly advantageous for the development of a robust and high-quality method [30].

In addition to HPLC method optimization, one study used the AQbD approach in the search for the development of green analytical methods [30]. By incorporating the principles of green analytical chemistry into the AQbD approach, it is possible to ensure that an analytical method meets the intended performance while adhering to environmentally friendly specifications [30]. The versatility of AQbD was also demonstrated in studies that applied it to other techniques. Applications were identified for the development of methods in rheology [29], permeation [32], gel electrophoresis [35], and dissolution [34]. In particular, the dissolution study presented a direct critique of the traditional approach for the development of dissolution methods, as it is time-consuming and often results in ineffective and non-discriminatory methods [34]. A discriminative dissolution method is essential to identify potential impacts on the clinical performance of a drug caused by changes in formulation or process [40, 41].

In another work, the authors applied AQbD principles to develop a capillary electrophoresis method. The main goal was to replace an outdated analytical technology used in a marketed therapeutic monoclonal antibody. The acquired knowledge enabled the method switch, highlighting the importance of AQbD in the modernization and improvement of analytical methods [35]. Also focusing on complex analytical methods, Sena et al. (2023) used the AQbD approach in the development of an in vitro buccal mucosa permeation method, a technique that faces several challenges when attempting to predict the in vivo behavior of a drug, as a wide range of variables and conditions must be controlled and optimized [32].

A relevant study highlighted the growing importance of AQbD, as reflected in the ICH guidelines. However, it also pointed out that this systematic approach is the opposite of the approach seen in many of the published pharmacopeial methods. The authors explain that current compendial methods for analyzing fatty acids require critical derivatization steps for gas chromatography analysis, leading to long analysis times and high costs due to the use of gases (helium and hydrogen). Therefore, they proposed an alternative via liquid chromatography with an aerosol detector, applying the AQbD approach, which allowed mapping the critical parameters (e.g., column temperature, flow rate, gradient elution time) and critical method attributes (e.g., peak resolution, retention time), and establishing the MODR through Monte Carlo simulations, ensuring a robust method throughout its lifecycle [37].

### Distinctions Between the Steps Involved in AQbD

The analysis revealed that most of them did not address all the elements of AQbD, with varying numbers of stages involved in the implementation of this approach. In many studies [29–31, 33, 34], a streamlined construction of the ATP was carried out, in the form of simple text, with the goal and selection of CMAs that needed to comply with the defined ATP, followed by a risk assessment (often using the Ishikawa methodology) to classify and evaluate the CMPs (independent variables) that would affect the CMAs (dependent variables). Subsequently, preliminary tests or a screening DoE were conducted to fix some parameters and reduce the factors that went into the optimization DoE, which aimed to study the relationship between CMAs and CMPs. Based on the DoE results, the MODR was constructed to meet the criteria established for each CMA simultaneously, and finally, a validation stage was performed to ensure the method’s performance. These works did not explore the construction of the ATP, elaborating on the targets, justifications, and defining acceptable measurement uncertainty; nor did they propose control strategies to ensure the method’s compliance throughout its lifecycle. In some cases, they merely mentioned certain AQbD elements in the text without presenting the results of that stage in the study’s discussion [31, 33, 35].

Some authors [15, 29, 42] provided a comprehensive set of information and data regarding the approach, offering supplementary details that are extremely helpful in understanding how AQbD was applied in method development. These studies serve as models for future work in this field. As discussed in Jackson et al. [44], the Analytical Target Profile (ATP) provides a structured framework for defining method performance requirements. In this context, some publications illustrate ATP construction through tabular formats to present analytical performance expectations clearly [32, 35]; these examples refer only to the organization of ATP elements and are not related to Critical Method Parameters (CMPs).

Simeoni et al. (2023) highlighted the importance of constructing the ATP while considering measurement uncertainty. To develop a meaningful ATP, a deep understanding of the product and process was necessary, enabling the definition of a clear purpose and performance requirements for the method within the overall control strategy, aiming to minimize patient risks. Measurement uncertainty was quantified using the Total Measurement Uncertainty (TMU) approach. The authors implemented performance controls and System Suitability Test (SST) criteria for the technology to ensure continuous verification of the method’s performance over time. This approach links the levels of the ATP, the technology-specific output requirements, and the SSTs, ensuring that unexpected changes in method parameters are detected and do not affect the performance requirements of the reportable result [35].

Santana, Rostagno, and Breitkreitz (2023) constructed their ATP by defining the objective and performance criteria of the method, ensuring confidence in the reported result and compliance with the specification. The ATP was: to develop a method capable of differentiating critical species based on their secondary metabolite profiles and determining a working range to accurately quantify target compounds within a pre-established total error of ± 20% for quality control. This is an example of an ATP where we do not see the definition of a specific analytical technique but rather criteria for selecting the most appropriate technique [42].

In another study, the ATP focused on the definition of CMAs, without describing the objective of the analytical method, nor its target and performance criteria. Patil et al. (2023) constructed the ATP by defining the following CMAs for an HPLC-based quantification method for esculin: retention time, theoretical plates, and peak symmetry. Afterward, the authors defined the CMPs that affect the ATP and evaluated the effects of these CMPs on the ATP using a DoE methodology called central composite design. The definitions of ATP and CMA were somewhat blended in this study [33].

A relevant study exemplified the careful construction of the ATP, defining the objective and expected performance of the method through target measurement uncertainty [36]. The ATP was: to develop a method capable of quantifying two active ingredients (hydrochlorothiazide and losartan) in the presence of their major impurities, with a global standard uncertainty below 1.25%, which was calculated based on the upper and lower specification limits. The study emphasized that information on uncertainty should be considered when evaluating compliance/non-compliance of the tested product or batch, due to the risk of false decisions. In the same study, after validating the optimized analytical method for content and degradation products, measurement uncertainties were assessed using a top-down approach. Additionally, multivariate guard bands were defined, ensuring a total false decision risk below 5% [36].

Monte Carlo simulations have proven to be the most commonly used method for estimating model uncertainty, thus providing an adequate level of assurance that the CMA specifications will be met in the MODR [43]. In the work of Santana, Rostagno, and Breitkreitz (2023), the authors employed the Monte Carlo method to incorporate the uncertainties of the CMPs into the MODR to ensure the robustness of the method. Furthermore, the study highlights that when defining the MODR, establishing the ranges for the CMPs in is a way that simultaneously meets the criteria for each CMA is not an easy task [42].

The work reported by Lopes et al. (2024) aimed to establish the ATP based on the previously defined Quality Target Product Profile (QTPP) for the drug product. The ATP involved: an efficient and selective HPLC method to quantify the active ingredient within a defined specification range, with an acceptable maximum standard uncertainty, in the presence of related substances, each with a maximum specification and an acceptable maximum standard uncertainty as well. Moreover, in this study, both Monte Carlo simulations and DoE results were used to construct the MODR. The method validation also contributed to the measurement of uncertainty estimation through a top-down. Additionally, the authors applied a decision rule using guard bands to adjust the specification to a more restricted acceptance zone, thus reducing the risk of a false decision being made. Finally, the authors designed a control strategy for the method by defining SST of the analytical procedure system [15].

One study addressed the importance of re-evaluating the risk analysis conducted at the beginning of the analytical method development by AQbD. The author used a risk assessment methodology, Failure Modes, Effects, and Criticality Analysis (FMECA), to revisit the high-risk factors identified during the development phase, proposing more effective preventive actions. The research demonstrates that as the understanding of the method evolves, it is crucial to update the previously established risk levels, especially for method-specific variables that have a significant impact on the method’s CMAs [29]. Sena et al. (2023) also discuss this update of the risk matrix, considering the method’s lifecycle, where changes may be necessary to support innovation and continuous improvement [32].

### Literature Remarks

The literature analysis indicates that the application of AQbD has expanded beyond the strictly regulated environment of the pharmaceutical industry, with an increasing presence in academia and, to a lesser extent, in collaborative initiatives between the two sectors. This expansion demonstrates the recognition of AQbD’s potential for developing more robust, optimized, and reliable analytical methods, even as an alternative to traditional techniques like HPLC. It was observed that AQbD enables a better understanding of the factors involved and their influence on method attributes, allowing for the development of optimized methods with a lower risk of making false decisions regarding product conformity. The data show a trend toward greater uniformity in terminology and acronyms over the years. However, there is considerable variability in how AQbD elements are implemented, the number of stages involved, and the content of these elements.

Although key stages, such as the definition of the ATP, identification of CMAs and CMPs, application of DoE, establishment of the MODR, and validation, are frequently cited, their practical application varies widely in terms of depth and detail. This reveals the absence of a consolidated and uniform standard for implementing AQbD, as also pointed out in the literature by Bastogne et al. (2022), who emphasize the need for a “gold standard” guide to steer researchers and professionals in the field [21].

It was possible to assess that the description of this element varied across many studies, with many not defining the target to be achieved with the analytical method and even fewer establishing decision rules for conformity evaluation. On the other hand, almost all the studies developed analytical methods that were operable within the parameters limits of their MODR, capable of providing quality results and ensuring regulatory flexibility. However, there is little detail on how this flexibility translates into practical actions, and few defined appropriate control strategies based on the defined MODR. From this, it is possible to plan action plans to be applied to the CMPs to ensure that the analytical method meets performance requirements and complies with pre-established specifications [44]. Additionally, lifecycle management of the analytical method was an aspect rarely discussed, despite its relevance in the context of AQbD.

## Conclusion

This study analyzes the implementation of AQbD in Southeast Brazil’s pharmaceutical industry. Although 88.3% of surveyed companies are familiar with AQbD, only 50% have started adopting it, mostly in early stages using tools like DoE and risk assessment. Key elements such as defining the MODR and lifecycle monitoring are rarely applied. Barriers include limited technical resources, lack of trained personnel, cultural resistance, and the absence of clear regulatory requirements. The literature highlights the potential of AQbD to improve robustness and process understanding but also points to inconsistencies in its application, especially in defining the ATP and establishing control strategies. Recent studies show gradual progress through the adoption of advanced tools like guard bands and uncertainty assessment. Full implementation of AQbD depends on stronger collaboration among industry, academia, and regulatory agencies, along with improved training and national guidelines to support its broader adoption.

## Supplementary Information

Below is the link to the electronic supplementary material.

Supplementary Material 1

## Data Availability

No datasets were generated or analysed during the current study.

## Declarations

### Consented for Publication

All authors have approved the final article and consented to publication.
