# Supplementary material for: A Survey on the Implementation of Analytical Quality by Design in Method Development in the Pharmaceutical Industries of Southeast Brazil: Challenges and Opportunities
Source: Ther Innov Regul Sci. 2026 Feb 24;60(3):879–92. doi: 10.1007/s43441-026-00930-2 (PMC13110218; doi:10.1007/s43441-026-00930-2)
Supplement: Supplementary file 1 — Supplementary Material 1 [file 43441_2026_930_MOESM1_ESM.docx]

**Supplementary information**

**QUESTIONNAIRE**

**PART 1 – TCLE**

1. Email: ______________________
2. Do you agree to participate in this research?

⃝ Yes, I have read the Free and Informed Consent Form and agree to participate in the research.

⃝ I do not wish to participate in the research.

**PART 2 – GENERAL INFORMATION ABOUT THE COMPANY**

1. Sector in the company: __________________________________________________________
2. Is the company national or multinational?

⃝ National

⃝ Multinational

1. Does the company have a research and development (R&D) department in Brazil?

⃝ Yes

⃝ No

5.1. If yes, how long has the R&D sector existed in Brazil? (Write the number in years or, if you don’t know, write “I don’t know.”) ________

1. The company develops medicines (you can select more than one option):

⃝ Similar

⃝ Generics

⃝ Innovators

**PART 3 – CONCEPTUAL ASPECTS AND IMPLEMENTATION OF AQbD**

1. The company understands the *Quality by Design* (QbD) concept applied to analytical methods, also known as *Analytical Quality by Design* (AQbD)?

⃝ Yes

⃝ No (completed the questionnaire)

1. Is the company familiar with the ICH Q14 guideline?

⃝ Yes

⃝ No

1. Is the company familiar with the general chapter of the US Pharmacopeia <1220>?

⃝ Yes

⃝ No

1. Does the company use measures, methods, or tools from AQbD, or has it started implementing AQbD in the development, validation, and/or continuous monitoring of analytical methods?

⃝ Yes (continue to the next question)

⃝ No (go to question 14)

1. When did the company start using measures, methods or tools specific to the AQbD concept?

⃝ Before 2017

⃝ 2017 – 2022

⃝ 2022 - Current

⃝ I don't know

1. How would you classify the degree of implementation of AQbD for the development of analytical methods (for content, dissolution, among others) in the company?

⃝ None, we do not have any type of activities, but we are analyzing this possibility.

⃝ Low, we apply some measures/tools in few projects.

⃝ Medium, we have already developed some methods applying AQbD .

⃝ High, we use it systematically in our projects.

⃝ I don't know

1. What measures, methods or tools the company uses or is implementing (you can select more than one option).

⃝ Definition of the Analytical Target Profile (ATP) at the beginning of a product research and development project;

⃝ Definition of potentially critical method attributes and parameters;

⃝ Use of risk assessment tools;

⃝ Use of Design of Experimental (DoE;

⃝ Establishment of the Method Operable Design Region (MODR) also known as Design Space (DS).

⃝ Establishment of a control strategy for continuous monitoring throughout the life cycle.

⃝ Use of false decision risk reduction approaches to assess product compliance with the specification (e.g., determination of measurement uncertainty, use of guard bands, among others).

1. How would you rate your company's perception of the importance of AQbD , considering the budget level and/or number of dedicated human resources.

⃝ Unimportant, without budget or dedicated human resources.

⃝ Little important, little budget and/or dedicated human resources.

⃝ Important, relevant budget and/or dedicated human resources.

⃝ Very important, high budget and/or dedicated human resources.

1. Which departaments of the company are involved or have implications with the application of AQbD ? (you can select more than one option)

⃝ Analytical Development Laboratory

⃝ Pharmaceutical Development Laboratory

⃝ Quality Control

⃝ Quality Management

⃝ Regulatory Affairs

⃝ Others

|  |
| --- |

1. What benefit( s ) has the implementation of AQbD brought or do you believe it could bring to the company? (you can select more than one option)

⃝ Greater flexibility/capacity for change;

⃝ Improvement of the quality of the final product ;

⃝ More robust and systematically developed methods;

⃝ Greater knowledge about the product, the development process and the interactions between analytical variables;

⃝ Reduction in the risk of out-of-specification (OOS) and trend (OOT) results;

⃝ Ensure greater product compliance with reduced risk for the consumer (patient) and for the producer (pharmaceutical industry);

⃝ Greater ease in transferring methodologies between laboratories.

⃝ Others:

|  |
| --- |

1. What are the difficulties in implementing AQbD (or the reasons for its non-implementation)? (you can select more than one option)

⃝ Difficulties in understanding AQbD concepts ;

⃝ Uncertainties about how to actually apply the AQbD principles ;

⃝ Difficulty in quantifying the benefits of AQbD and the return on investment;

⃝ Difficulty in allocating time, financial resources (purchase of software and equipment) and qualified personnel;

⃝ Company management does not understand or support AQbD ;

⃝ Non-mandatory implementation of AQbD in Brazil;

⃝ Culture change for the professionals involved.

⃝ Others:

|  |
| --- |

1. Comments or suggestions:

|  |
| --- |
